# Effect of silver diamine fluoride on the bond durability of normal and carious dentin

**DOI:** 10.4317/jced.56303

**Published:** 2020-05-01

**Authors:** Maryam Firouzmandi, Mina Mohaghegh, Maedeh Jafarpisheh

**Affiliations:** 1 Assistant Professor, Oral and Dental Disease Research Center, Department of Operative Dentistry, School of Dentistry, Shiraz University of Medical Sciences, Shiraz, Iran; 2 Assistant professor, Department of prosthodontics, School of Dentistry, Shiraz University of Medical Sciences, Shiraz, Iran; 3 Undergraduate Dental Student, Student Research Committee, School of Dentistry, Shiraz University of Medical Science, Shiraz, Iran

## Abstract

**Background:**

Silver diamine fluoride (SDF) has attracted attention because of its clinical success in arresting dentin caries. It has been shown that it can inhibit matrix metalloproteinases and cysteine cathepsins. These two properties might be beneficial in bonding to caries-affected dentin (CAD). Therefore, the present study aimed to investigate the effect of SDF on the durability and bond strength to the CAD.

**Material and Methods:**

Forty-eight third molars with occlusal caries were used. The roots were cut, and the occlusal enamel was removed. The CAD area surrounded by the normal dentin (ND) was exposed. All the specimens were bonded with an etch-and-rinse adhesive, but half of them were treated with SDF after acid etching (NT or SDF). Two cylinders of composite (0.9 mm in diameter and 0.7 mm in height) were built-up on each specimen, one on the CAD and the other on the ND area. The specimens in each group (NT-ND, NT, CAD, SDF-ND, SDF-CAD) (n=24) were equally divided into two subgroups. One subgroup was tested for microshear bond strength 24 hours after bonding (T0), and the other subgroup was tested after six months of water storage (T6). The bond strength data were analyzed using three-way ANOVA. Subgroup analysis was performed using independent samples t-test. Fracture patterns were also evaluated.

**Results:**

At T0, the bond strength of NT-ND was higher than that of NT-CAD (*p*<0.00), but the bond strength of SDF-ND and SDF-CAD were not different (*p*=0.77). Comparison of the bond strength of the groups between the two time intervals yielded the following results: NT-ND-T0> NT-ND-T6 (*p*=0.04), SDF-ND-T0=SDF-ND-T6 (*p*=0.39), NT-CAD-T0=NT-CAD-T6 (*p*=0.51) and SDF-CAD-T0>SDF-CAD-T6 (*p*<0.00).

**Conclusions:**

The SDF treatment increased the bond strength to CAD but did not affect the bond strength to the ND. SDF hindered the decrease in the bond strength to the ND caused by aging. However, the effect of SDF on increasing the bond strength to CAD disappeared after aging.

** Key words:**Silver diamine fluoride, caries-affected dentin, bond strength.

## Introduction

Minimally invasive dentistry aims to arrest caries or remove as little tooth structure as possible. Based on this concept, during treatment of dentinal caries, the aim is to remove the outer layer of infected caries and treat the inner affected dentin (1). Several recent studies have investigated the bond strength of adhesives to affected dentin and have recommended keeping affected dentin in restorative procedures (2,3).

Degradation of the hybrid layer influences the durability of dentin bonding. The resin component of the hybrid layer is prone to degradation by hydrolysis (4). Degradation of the collagen matrix is induced by cysteine cathepsins and matrix metalloproteinases (MMPs) (5). The MMPs are endogenous proteases that are trapped in the dentinal matrix in the form of latent zymogens (pro-MMPs). MMPs have an important role in the enzymatic degradation of extracellular matrix components (5). Cysteine cathepsins have been detected in carious dentin. They can activate MMPs or directly degrade type I collagen in the dentin (6). Both of these enzymes might become active due to exposure to acidic environments during the caries process or acid etching. These proteases hydrolyze peptide bonds in the collagen molecules at the adhesive–dentin interface (6,7). They are responsible for resin–dentin bond degradation and can jeopardize the bond durability. Several studies have utilized chlorhexidine (8), proanthocyanidin (9) and carbodimide (10) to inactivate these proteases during the dentin bonding procedure and increase the durability of the bond to dentin.

MMPs are also known to be involved in the dentin matrix destruction during the caries process; thus, MMP inhibitors might be effective in arresting caries. In the past, bacterial proteases were considered to be responsible for the dentin matrix degradation, but it has been shown that these enzymes become inactive by the low pH during demineralization (7).

Silver diamine fluoride (SDF) is an affordable, effective, safe and easy-to-use caries arresting agent (11). Clinical studies have shown that SDF decreases the rate of demineralization in the tooth structure (12), arrests and prevents coronal caries in deciduous teeth (12) and root caries in permanent teeth (13). Also, SDF can inhibit the growth of cariogenic bacteria in the biofilm (14). Silver compounds penetrate into the dentinal tubules, invading the cariogenic microorganisms (11). In addition, they can harden the carious lesion (5), inhibit degradation of the collagen in demineralized dentin (15) and exert an inhibitory effect on MMPs (7) and cysteine cathepsins (6). Therefore, SDF can be considered as a pretreatment when bonding to CAD to remineralize and arrest caries, destroy residual bacteria, and promote the bond durability.

Previous studies have investigated the effect of SDF on the bond strength of glass-ionomer (16), etch-and-rinse and self-etch adhesive to normal dentin.(17) However, there is no study to evaluate the effect of SDF on the stability of the bond to the CAD. Therefore, this study aimed to investigate the effect of SDF on the immediate and mid-term bond strength to either normal or affected dentin. The null hypothesis ran as follows: Neither the SDF application nor the water storage period would have any effect on the bond strength to caries-affected and normal dentin.

## Material and Methods

-Specimen Preparation

Carious human third molars without previous restorations that were extracted for surgical reasons were used in this study. The study protocol was approved by the university ethic committee. Forty-eight teeth with occlusal caries extending approximately two-thirds into the dentin were selected through periapical radiography. Immediately after extraction, the teeth were thoroughly washed and stored in 0.5% chloramine-T solution at 4°C and used within four weeks.

The roots were cut at the CEJ level, and the occlusal enamel was trimmed using a low-speed rotary grinding machine under water coolant to provide a uniform flat dentin surface, perpendicular to the long axis of the teeth. The CAD was differentiated with the aid of caries-detecting dye in addition to visual and tactile methods. 1% acid red dye in propylene glycol (Kuraray Medicine Inc, Tokyo, Japan), as a caries detector, was applied on the trimmed tooth surfaces and rubbed with a micro-brush. After two minutes, the specimens were washed under running water. The external layer of the lesion that was stained red was removed with a round carbide bur to reach the light pink zone. The excavated area was inspected visually with the North Carolina Dentin Sclerosis Scale, which yielded a category of ‘4’ in which the dentin exhibits a glassy appearance and a dark yellow or slightly brownish color, with the bulk of the dentin exhibiting transparency (18). All the specimens were finished using 600-grit SiC paper for 10 seconds under running water. Finally, 48 specimens with internal caries (CAD) surrounded by the normal dentin (ND) were ready to be used.

-Bonding Procedures

Half of the specimens, which served as the controls (NT), were etched with 37% phosphoric acid (3M ESPE, Neuss, Germany) for 15 seconds, washed for 15 seconds, and then mildly blot-dried with cotton pellets. Subsequently, two layers of the bonding agent (Adper Single Bond2, 3M ESPE, Neuss, Germany) were applied according to the manufacturer’s instructions. Each specimen was light-cured for 10 seconds with a light-curing unit (Blue LEX 1200W, MONITEX, San-Chong City, Taipei, Taiwan) at a light intensity of 700–800 mW/cm2, which was checked with a radiometer (Demetron LED Radiometer, SDS, Kerr, Orange, CA, USA). The other half of the specimens were prepared according to the protocol mentioned aboved, but after acid etching, 30% SDF solution (Ancarie Cariostatic, Maquira Dental Product, Maringa, PR, Brazil) was applied on the surface of the specimens using a micro-brush. After three minutes, the surface of each specimen was carefully washed for 30 seconds. Plastic tubes (polyethylene tubes), measuring 0.9 mm in internal diameter and approximately 0.7 mm in length, were used for composite resin packing . A composite resin (Filtek Z250, 3M ESPE, USA) of A1 shade was applied and condensed into the tubes to ensure that there were no air bubbles inside. Each composite resin cylinder was light-cured for 40 seconds. The tubes were cautiously cut using a scalpel blade and then removed. Each micro-cylinder diameter was checked using a digital caliper (Mitutoyo 500-1710-6, Digimatic Caliper, Mitutoyo Corp, Tokyo, Japan(. On each specimen, two composite resin cylinders were built-up, one on the CAD area and the other on the ND.

The specimens in each experimental group (NT-ND, NT-CAD, SDF-ND, SDF-CAD) were equally divided into two subgroups (n=12). One subgroup was stored in distilled water for 24 hours at 37°C before microshear bond strength testing (T0). The other subgroup was stored in distilled water at 37°C for six months (T6).

-Microshear Bond Strength (µSBS) Testing

All the specimens were subjected to a µSBS test. Each specimen was mounted in a universal testing machine (Zwick/Roel, Germany), and microshear force was applied at a crosshead speed of 0.5 mm/min until debonding occurred, and the maximum load to failure was recorded. The bond strength was reported by dividing the load at failure by the bonded cross-sectional area and reported in MPa. After the bond strength testing, the failure pattern of the specimens was analyzed under a stereomicroscope at ×40 magnification to assess the failure modes which were classified as adhesive, cohesive in dentin, cohesive in composite resin or mixed.

-Statistical Analysis

The bond strength data were analyzed using three-way ANOVA. The main factors consisted of substrate type, treatment and time. Subgroup analysis was performed using independent samples t-test. The significance level was set at *p*<0.05.

## Results

Table 1 presents the results of the µSBS test. The highest bond strength was recorded in the NT-ND-T0 group. The lowest mean was seen in the SDF-CAD-T6 group. The effects of substrate type and time on the bond strength were significant (*p*<0.00), but the effect of treatment was not significant (*p*=0.21). The interaction of the three factors was not significant.

**Table 1:**
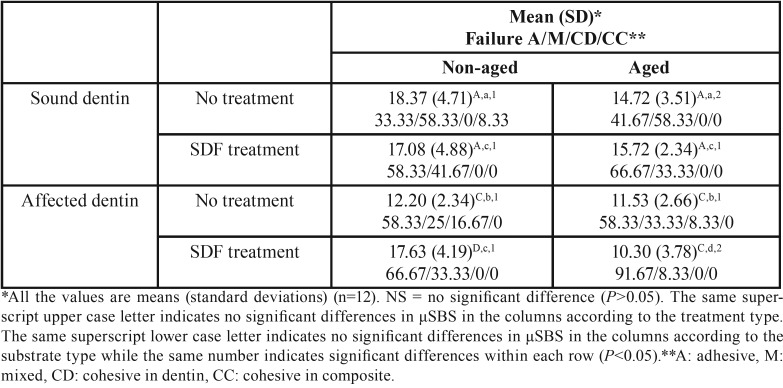
Means (MPa) and standard deviations of µSBS in the groups after 24 hours (T0) and 6 months (T6) and their fracture modes (%).

At T0, without the SDF treatment, the bond strength to the ND was higher than that to the CAD (*p*<0.00). However, with the SDF treatment, the bond strength to both substrates was not significantly different (*p*=0.77). At T6 with or without the SDF treatment, the bond strength to the ND was higher than that to the CAD (*p*=0.02 and *p*<0.00, respectively).

The SDF treatment of the ND did not affect the bond strength, neither at T0 (*p*=0.52) nor at T6 (*p*=0.42). The SDF treatment of the CAD increased the bond strength at T0 (*p*<0.00) but not at T6 (*p*=0.37).

In the groups without SDF treatment the bond strength to the ND reduced with aging (*p*=0.04). However aging process did not affected the bond strength to the CAD (*p*=0.51). In the SDF treatment groups the bond strength to the ND did not affected by aging (*p*=0.39), but in the CAD, the bond strength decreased after aging (*p*<0.00).

## Discussion

**Figure 1:**
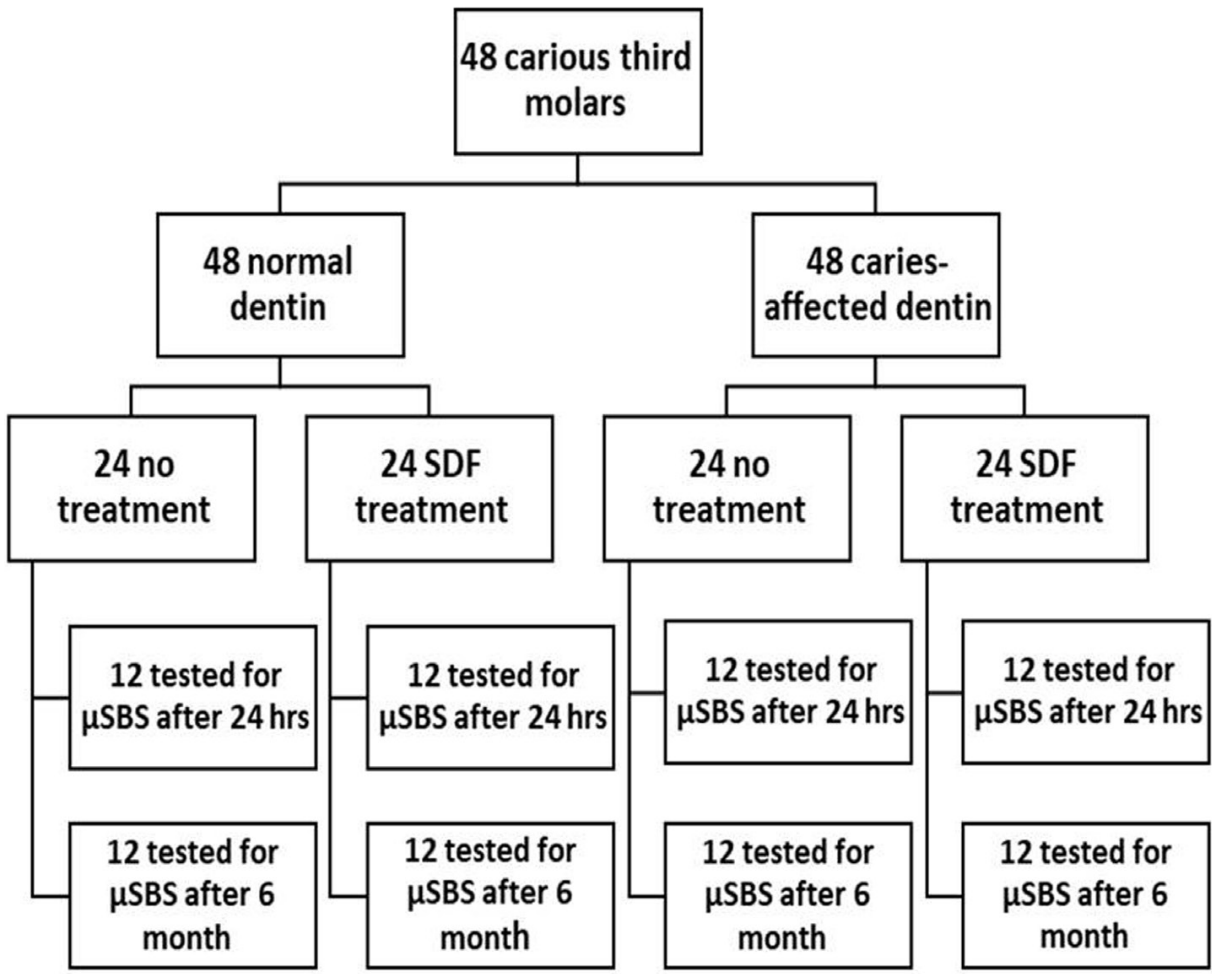
Flowchart of the experiment design.

The results of the present study showed that the substrate type and storage time affected the bond strength. Therefore, the null hypothesis of the study was refuted in part. Regardless of the storage time, the bond strength to the ND was higher than that to the AD, consistent with several previous studies (8,18,19). A recent meta-analysis also concluded the same (20). The lumens of dentinal tubules in the CAD are filled with carious crystals; thus, resin infiltration into the tubules is restricted (18,21), (Fig. 1). The porosity of intertubular dentin increases in the CAD as a result of demineralization during the caries process. This might permit the deeper etching of the intertubular dentin and more collagen exposure. The bond strength to the CAD might decrease because of irregular and incomplete hybridization of the etched dentin layer (22). The lower mechanical properties of the CAD due to demineralization can be accounted for the lower bond strength (22,23). Some studies contradicted this finding, which can be explained by different adhesive systems used (24-26). SDF application on the ND did not affect the bond strength at T0, but significantly increased the bond strength to the CAD. The proposed chemical interaction of SDF with hydroxyapatite is as follows (15): Ca10(PO4)(OH)2 ‏+ Ag(NH3(2F, 20Ag3PO4 +‏ 30CaF2 ‏+ NH4OH.

Studies have shown that SDF can remineralize the demineralized dentin (14,15). Therefore, the over-etching problem caused by etch-and-rinse adhesives on the CAD could be compensated by the SDF application, and the formed hybrid layer might be less porous and irregular. SDF, with its remineralizing capacity, can improve the mechanical properties of the CAD (27). This can, in return, increase the bond strength. Quock *et al.* also concluded that the bond strength of the etch-and-rinse adhesive to the ND was not influenced by the SDF treatment (17) but they applied SDF before acid etching. However, this result is contradictory to a previous study that reported the SDF application reduced the bond strength to both the ND and CAD (28). The adhesive used in that study was an MDP-containing self-etch adhesive, which can chemically bond to calcium and phosphate ions. As illustrated in the reaction formula mentioned above, silver phosphate and calcium fluoride precipitates are formed by the SDF application. Therefore, calcium and phosphate ions might not be available for chemical bonding.

It has been proposed that the collagenolytic activity of the CAD is higher than the ND because of the substantial activity of MMPs and cysteine cathepsins (21). Therefore, a higher rate of bond degradation is expected on the CAD. However, six months of water storage in the no-treatment groups adversely affected the bond strength to the ND, but the bond strength to the CAD was not influenced. Consistent with this result, another study reported CAD bond stability over time (9). A previous study which evaluated the durability of the µSBS to the ND and CAD reported that aging decreases the bond strength to both substrates (24). In that study, µSBS to the CAD without aging was higher than that in the current study (21.73 vs. 12.20), but after aging, the results were comparable (9.97 vs. 11.53). The subjectivity of the CAD definition might justify this difference.

The bond stability to the CAD observed in the present study might be related to the lower immediate bond strength and not to the lower decrease over time. Other studies showed reduced bond strength to the CAD with different adhesive systems after water storage (21,29). However, they used artificially created CAD and 12 months of aging. There is no consensus in the literature on the optimal water storage time to provoke the hybrid layer degeneration by endogenous proteases. Although Leinz *et al.* concluded that the artificially created CAD, both by pH cycling method or microbiologic method, is suiTable for bond longevity studies (21), natural CAD might have different adhesive behaviors. Tubular lumens in the natural CAD are filled with acid-resistant mineralized crystals. These precipitates reduce the dentin permeability and water conductance. Sealed dentin tubules might restrict water transfer to the adhesive interface during the storage period. In the ND group, acid etching removed the smear layer; therefore, during the storage period, the dentinal tubules might act as water-filled canals conducting water to the adhesive interface. Water can cause hydrolysis of the denuded collagen at the base of the hybrid layer and adhesive resin, facilitating the bond degradation.

The SDF application on the ND restricted the effect of water storage on the bond strength. On the CAD, a significant reduction of bond strength after water storage was shown in the SDF treated group. MMPs are involved in the digestion of all the extracellular matrix molecules, including native and denaturated collagen. MMP-8 cleaves collagen molecules into smaller peptides. MMP-2 and MMP-9 further degrade these peptides (7). Significant inhibition of MMP-2, MMP-8 and MMP-9 by 30% and 38% SDF was recorded in a previous study. However, the inhibitory effect of 38% SDF was stronger (7). This inhibitory effect can stop the degradation of the organic substance in the dentin. MMP inhibitors could be considered to reduce the progression of dentinal caries (7). SDF can also inhibit cysteine cathepsins. These enzymes are active and stable in acidic pH (6). Recent studies have described them as the key elements in the activation of MMPs and also collagen degradation (6). The percentage of contribution of MMPs and cysteine cathepsins in bond degradation is not clear and resin hydrolysis, as a result of simple water storage, should not be overlooked. On the CAD, the SDF application led to a significant increase in the bond strength, which was attributed to the remineralizing effect of SDF and improved mechanical properties of CAD. The water storage of the specimens might cause the silver nitrate and calcium fluoride compounds to be washed out, and the µSBS specimens’ form might have facilitated the dissolution process. The high porosity of the bond interface in the CAD groups might aggravate the situation.

In the current study, mixed failure pattern could be correlated with the relatively higher bond strength. The dominance of adhesive failures in the aged groups was in favor of reduced bond strength. More adhesive failures observed in the SDF-treated groups might be a possible indicator of lower quality of the hybrid layer, which is to be investigated in future studies. Moreover, the cohesive failure pattern observed in the groups bonded to the CAD could have been due to the inherent weakness of the carious dentin.

## Conclusions

SDF treatment increased the bond strength to the CAD but did not affect the bond strength to the ND. Aging reduced the bond strength to the ND but did not affect the bond strength to the CAD. The decrease in the bond strength to the ND caused by aging was hindered by SDF. However, the effect of SDF on increasing the bond strength to the CAD disappeared after aging.
